# The Erasmus+ EUMOVE project—a school-based promotion of healthy lifestyles to prevent obesity in European children and adolescents

**DOI:** 10.1093/eurpub/ckae113

**Published:** 2024-07-29

**Authors:** Abel Ruiz-Hermosa, Mairena Sánchez-López, José Castro-Piñero, Alberto Grao-Cruces, Daniel Camiletti-Moirón, João Martins, Jorge Mota, Andrea Ceciliani, Marie Murphy, Anne Vuillemin, David Sánchez-Oliva, EUMOVE Consortium, Tomás García Calvo, Inmaculada González Ponce, Juan José Pulido González, Francisco Miguel Leo Marcos, Pedro Antonio Sánchez Miguel, Diego Muñoz Marín, Javier Sevil Serrano, Miguel Ángel Tapia Serrano, Rocío Izquierdo Gómez, Julio Conde Caveda, Carmen Padilla Moledo, Vicente Martínez-Vizcaíno, Carlos González Morcillo, Palma Chillón Garzón, Marcos Onofre, Adilson Marques, Tiago Ribeiro, Paula Silva, Paula Santos, Andreia Pizarro, Karine Corrion, Jean-Marie Garbarino, Laura Dallolio, Alice Masini, Sofia Marini, Angela Carlin, Sinead Connolly, Nuno Ferro, Felix Bolaños, João Costa, Dusan Pjevac, Marion Fournier, Raffaela Mulato, Stephan Riegger

**Affiliations:** Social and Health Care Research Center, Universidad de Castilla-La Mancha, Cuenca, Spain; ACAFYDE Research Group, Faculty of Sports Sciences, University of Extremadura, Cáceres, Spain; Social and Health Care Research Center, Universidad de Castilla-La Mancha, Cuenca, Spain; Faculty of Education, Universidad de Castilla‐La Mancha, Ciudad Real, Spain; GALENO Research Group, Department of Physical Education, Faculty of Education Sciences, University of Cadiz, Puerto Real, Spain; Instituto de Investigación e Innovación Biomédica de Cádiz (INiBICA), Cadiz, Spain; GALENO Research Group, Department of Physical Education, Faculty of Education Sciences, University of Cadiz, Puerto Real, Spain; Instituto de Investigación e Innovación Biomédica de Cádiz (INiBICA), Cadiz, Spain; GALENO Research Group, Department of Physical Education, Faculty of Education Sciences, University of Cadiz, Puerto Real, Spain; Instituto de Investigación e Innovación Biomédica de Cádiz (INiBICA), Cadiz, Spain; Pedagogical Laboratory, Faculty of Human Kinetics, University of Lisbon, Portugal; Centro de Investigação em Actividade Física, Saúde e Lazer, Universidade do Porto, Porto, Portugal; Department for Life Quality Studies, University of Bologna, Bologna, Italy; Centre for Exercise, Physical Activity, Medicine and Health, Ulster University, Jordanstown, Northern Ireland, United Kingdom; Laboratoire Motricité Humaine Expertise Sport Santé (LAMHESS), Université Côte d’Azur, Nice, France; ACAFYDE Research Group, Faculty of Sports Sciences, University of Extremadura, Cáceres, Spain; GALENO Research Group, Department of Physical Education, Faculty of Education Sciences, University of Cadiz, Puerto Real, Spain; Instituto de Investigación e Innovación Biomédica de Cádiz (INiBICA), Cadiz, Spain

## Abstract

The aim of this study was to describe the goals, activities, and methods of EUMOVE project in developing a set of resources targeting both primary and secondary schools that allow the entire educational community to promote healthy lifestyles (HL). The EUMOVE project is an Erasmus+ program based in the Creating Active Schools (CAS) framework. The project lasted 3 years and was developed by 14 academic and non-governmental institutions from Spain, Portugal, France, Italy, and the UK. EUMOVE was divided into three phases. In the first phase, several work packages were carried out aimed to ensure the coordination/management of the project activities. In the second phase, seven educational resources strengthened by rigorous scientific research were developed to promote HL from schools. During the last phase, all the resources were disseminated through scientific seminars, workshops with families and teachers, and the online promotion by the non-governmental institutions of each country. The EUMOVE project developed and promoted a smartphone APP, video/activity repository of physically active lessons, active break virtual platform, learning units, and parent/teachers/school-leaders’ guidelines to promote physical activity, active commuting, active school playgrounds, healthy diet, and sleep habits. To our knowledge, EUMOVE is the first European project to provide a set of practical tools based on scientific evidence to help schools or future school-wide interventions implement a paradigm shift based on the CAS framework for the promotion of HL. Future research will need to investigate the implementation, effectiveness, and scalability of this proposal.

## Introduction

The childhood overweight/obesity has become one of the most serious public health problems in Europe [1], leading an increased incidence of non-communicable disease in adulthood [2]. According to a recent meta-analysis [1] that included 28 European countries, the prevalence of overweight/obesity from 2011-to-2016 in 2- to 13-year-old children was 21.3%. Moreover, the estimates for the future are not encouraging, as more than 200 million schoolchildren are projected to be obese by 2030 [3].

Although obesity is the result of the combination of several multidimensional factors, physical inactivity and sedentary behaviors are the main drivers [4]. It is not surprising, therefore, that the European schoolchildren have become more sedentary [5] and only one-third meet the recommendation of at least 60 min/day of moderate-to-vigorous Physical Activity (PA) [6]. Furthermore, exposure to unhealthy dietary and poor sleep habits (SH) could also be driving this trend in Europe [7–9].

Given youth spend most of their time in school, the school-day is an ideal context for obesity prevention through the promotion of PA and healthy lifestyles (HL) [10, 11]. However, previous research has found that the school day is the most sedentary period of the day for schoolchildren [12]. Moreover, although several studies have demonstrated that school-based PA programs have positive effects to increase PA, recent reviews have shown inconclusive results [10, 13]. Similarly, a meta-analysis focused on school-based obesity prevention programs have demonstrated limited efficacy of decreasing schoolchildren obesity [14]. This may result from the difficulty of designing feasible and sustainable interventions, as well as the lack of a comprehensive approach involving the whole school [10, 15, 16].

To reverse this situation, several studies [16, 17] and the World Health Organization (WHO) [11] have published guidelines for adequate promotion of HL through schools. These recommendations have highlighted that the most effective way to maximize the promotion of HL in the school day is to engage the entire educational community in a whole approach through multicomponent strategies [11, 16, 18].

In this line, the Creating Active Schools (CAS) Framework [16] is a method that suggests the development of policies/interventions by public administrations with competences in public health, education, and sports aimed at developing HL. Therefore, the school emerges as an excellent context for this purpose since the training of teachers will allow: to train students to be physically active; offer opportunities to be physically active; and motivate them toward the development of HL [19]. For this, the schools must contribute to the creation of physical/social environments that allow the promotion of HL through the participation of management teams, teachers, students, families, and other associations linked to education and public health. Through intervention in these groups, strategies/activities grouped into seven areas are implemented: events/visits, recess, physical education, curricular lessons, before/after school clubs, Active Commuting to School (ACS), and family/community [16, 19]. However, due to the novelty of this approach, more studies are needed to know the potential of the CAS. Furthermore, given that previous research has pointed out the need to design toolkit to facilitate and generate this paradigm shift [16, 19], it seems relevant to develop a set of resources based in these seven opportunities.

The EUMOVE project follow CAS framework to develop a set of educational resources based on scientific evidence: physically active lessons (PAL) [13, 20], active breaks (AB) [21, 22], recess [23, 24], ACS [25, 26], extracurricular HL [27], parents [28, 29], and school environment [13, 16]. Thus, the purpose of this paper is to describe the goals, activities, and methods of an Erasmus+ program (The *EUMOVE project*) based in the CAS framework for promote HL and preventing obesity in European children and adolescents (6–17 years old). The objectives of this project were developed and disseminate a practical toolkit that allow the entire educational community, future school-based interventions, and stakeholders to promote HL in children and adolescents.

## Methods

### Study design

The EUMOVE project (“*Let’s move Europa: school-based promotion of HL to prevent obesity*”) is an Erasmus+ program co-financed by the European Union (Ref.622242-EPP-1-2020-1-ES-SPO-SCP) to develop and disseminate resources for school-based interventions targeting both primary and secondary schools (6–17 years old) with the aim of promoting HL and preventing obesity in schoolchildren.

**Figure 1.**
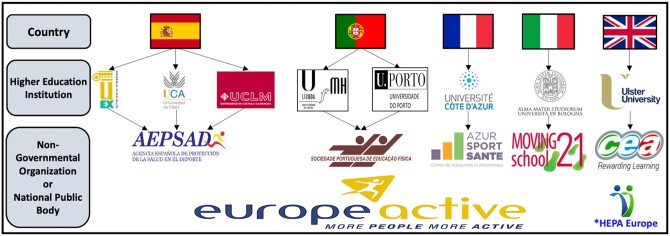
Participating partners for the EUMOVE Project. *Collaborator Network.

The project started in January 2021 and until December 2023, it was coordinated by the University of Extremadura (Spain) and was developed by 14 academic and non-governmental institutions from Spain, Portugal, France, Italy, and the UK. Each country had the participation of at least one public university focused on the joint design of resources and the coordination of the project, and at least one administration/association focused on the dissemination of the resources (Fig. 1). Additionally, the project had the collaboration as partners of EUROPEACTIVE for the dissemination throughout Europe. Moreover, the Health-Enhancing Physical Activity (HEPA) Europe network also participated in the dissemination process.

**Figure 2.**
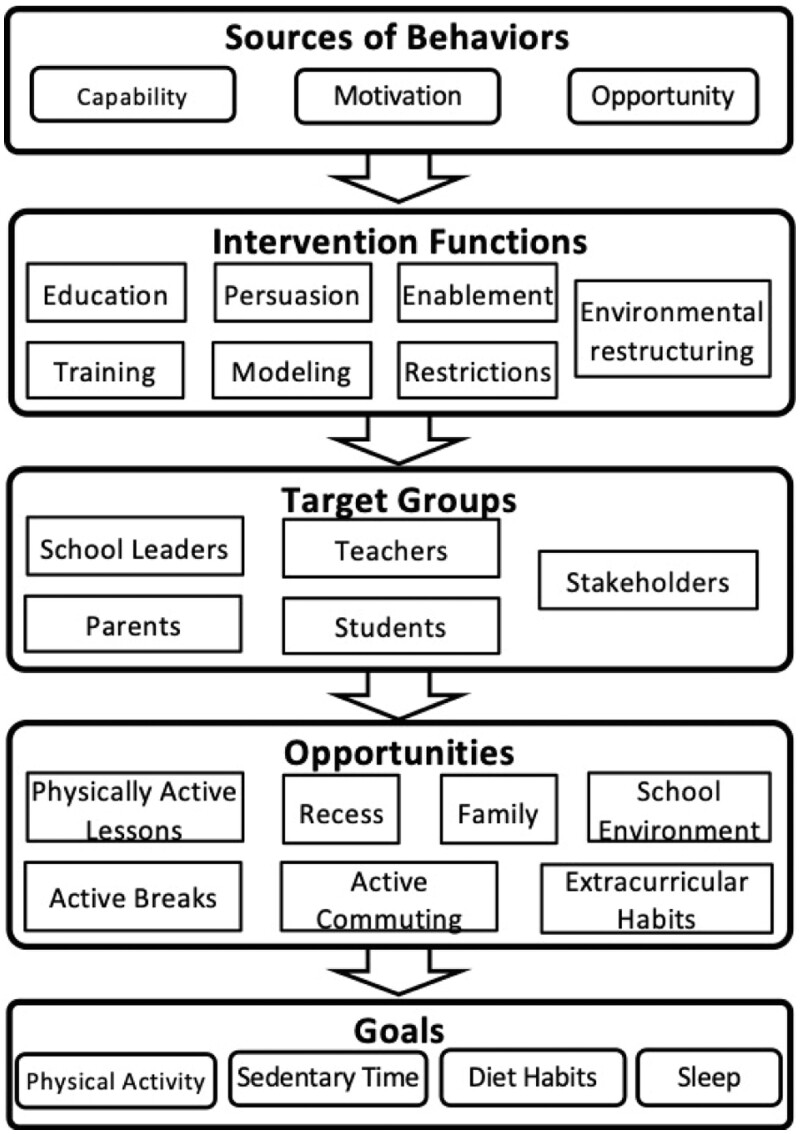
Design of EUMOVE project. Adapted from Daly-smith *et al*. [16].

The EUMOVE project was based on the CAS framework [16]. We used the three sources of behaviors (capability, motivation, and opportunity) for implementation across the five target groups (school leaders, teachers, children/adolescents, parents/guardians, and wider stakeholders) through different intervention functions (education, persuasion, enablement, training, modeling, restrictions, and environmental restructuring), by using seven opportunities (PAL, AB, recess, ACS, extracurricular HL, parents, and school environment) with the aim of increase PA, reduce sedentary time, and promote good diet and SH (Fig. 2). Therefore, since the CAS framework focuses on the promotion of PA using a whole-school adaptive sub-system as the main goal [16], this project followed a more holistic approach or adaptation by also including the promotion of good diet and SH.

### The EUMOVE project: description

The EUMOVE project was divided into three phases (Table 1):

**Table 1.** Project Design and EUMOVE’s work program

Phases of EUMOVE project		Main activities
**Phase 1: Project coordination and administration**		Initial Project Coordination
		Transnational meetings
		Project Administration and contact with EC
		Management and coordination of IOs
**Phase 2: Design and development of the practical toolkit and educational resources**	**1. EUMOVE Platform**	Design and creation of the EUMOVE Platform
		Inclusion of resources into the platform
	**2. EUMOVE smartphone APP**	Conceptualization of the APP
		Creation and design of the APP
	**3. Physically active lessons toolkit**	Intellectual design of activities
		Creation and production of the toolkit activities
	**4. Real time active breaks platform**	Intellectual design of activities
		Creation and design of the virtual avatar
		Creation and design of the active breaks platform
	**5. Active school commuting toolkit**	Intellectual design of strategies
		Creation and production of the toolkit strategies
	**6. Learning units about healthy lifestyles promotion**	Co-design Stage of the learning units
		Creation and production of the learning units
	**7. Parents toolkit about promoting healthy lifestyles**	Intellectual design of activities
		Creation and production of the toolkit activities
	**8. School leaders toolkit**	Intellectual design of activities
		Creation and production of the toolkit activities
**Phase 3: The dissemination plan**		Online dissemination
		Scientific seminars
		Teacher and parents workshops

EC, European Commission; IOs, Intellectual Outputs.

#### Project coordination/administration

During this phase work packages were carried out aimed to ensure the smooth coordination with the European Commission and participating sectors, the administrative/financial activities, as well as the correct develop, review and dissemination of the resources. The University of Extremadura, as the coordinating organization, oversaw the project both during and after its implementation and worked ongoing collaboration with all partners. For its, during the first two months of the project a guide was developed to be followed by all the partners, by detailing all the tasks, dates, objectives, responsibilities, and expecting procedures. Moreover, we prepared a Gantt chart with the aim to ensure all partners recorded the progress of the resources in which they were working on.

Additionally, to ensure the correct development of the project five transnational meetings were held in Spain, Italy, Lisbon, and France. These meetings implied the participation/connection of all the organisms/institutions of the project with the aim of evaluating the previous work, preparing the following work, and clarifying decisions. The main tasks developed are found in Supplementary material S1.

#### Design/development of educational resources

During this phase, a set of educational resources were developed from schools through the development of PAL, the implementation of AB, the promotion of ACS and recess, learning units (LU) of PA, healthy nutrition, and SH, and resources for families, school leaders, and educational authorities on the promotion of HL (including PA, healthy nutrition, and SH).

The resources were designed by the universities of the project. These organizations had experience on international projects and the project members were experts in school-based interventions to promote HL. Each university was responsible or participated in the organization of the development and evaluation of at least one of the resources generated. With the aim of getting more feedback, the preliminary version of each resource was reviewed by all organizations that were not involved in the development of this tool. This review also included the collaboration of active teachers belonging to the administrations/non-governmental associations of the consortium. Additionally, the universities responsible for each educational resource implemented it in the real context with the aim of considering the comments/suggestions of the students, teachers, and families. The leading organization of each resource and the main tasks are shown in Table 2.

**Table 2.** Leading organizations and participants in the development of EUMOVE educational resources

Educational resources	Leading organization	Participating organizations	Task
EUMOVE platform	University of Extremadura	All EUMOVE partners	- Development of the resources - Draft review by all partners - Definition of the resources - Translation to all languages^a^ - Upload to the Learning Platform
EUMOVE smartphone APP	University of Extremadura	University of Castilla-La Mancha	
Physically active lessons toolkit	University of Extremadura	University of Cádiz	
Real time active breaks platform	University of Castilla-La Mancha	University of Porto and University of Ulster	
Active school commuting toolkit	University of Cádiz	University of Porto and University of Ulster	
Learning units about healthy lifestyles promotion	University of Bologna	Moving School 21	
Parents toolkit about promoting healthy lifestyles	University of Lisbon	Sociedade Portuguesa de Educação Física	
School leaders toolkit	University of Extremadura	University of Porto and University of Ulster	

a All university partners will contribute with the toolkit translation in Spanish, English, French, Italian, and Portuguese.

All educational resources were free, multilanguage (Spanish, English, French, Italian, and Portuguese), and adapted to the educational curriculum of each country. Furthermore, these were based on the most current scientific evidence [16, 20, 21, 23, 25, 27, 28], were easy to implement, and did not require complex equipment. On the other hand, one of the premises of the project was that all resources were sustainable over time without the need for additional financing. Nevertheless, to ensure the sustainability, an economic distribution was allocated to guarantee the maintenance/technical support of the resources for at least 10 years.

A total of seven resources were generated that are hosted on the EUMOVE web (Supplementary material S2).

***EUMOVE web.*** An online portal was created to become the epicenter of activity of the EUMOVE project. This platform was open-access, included information about the project (goals, publications, contact, etc.), and was linked to the EUMOVE social networks, which allows updating project news/activities in both media. Furthermore, the platform aimed to upload the resources and was a key mechanism to ensure the online dissemination and sustainability of the full range of outputs of the project. Thus, this web was designed so that all educational community/stakeholders could consult/download the resources in a simple way.

***EUMOVE smartphone APP.*** It was a mobile-phone APP aimed at providing strategies to promote HL among students through three ways of connections. On the one hand, project researchers sent teachers’ practical information about strategies to promote HL from schools, information of EUMOVE resources, WHO guidelines, etc. On the other hand, project researchers sent information about strategies to promote HL among their children. For this, different flyers were designed and sent to families/parents through the APP, including recommendations for supporting their children to practice exercise, examples of healthy breakfasts/lunches/dinners, and recommendation for adequate SH. Finally, the APP connected teachers with parents for useful information about HL. In this line, each teacher could only contact with parents form their students, and they can use this tool for using strategies to promote PA, inform about practice options within the location, suggest the use of other EUMOVE resources, etc. Thus, these three forms of connection and the autonomous use of the APP will generate a set of resources and information of interest hosted in the same place that could ensure its sustainability. The user will be able to access the app online on multi-devices using either a mobile device or a computer. The APP is available in Google Play and Apple Store here.

***PAL toolkit.*** The aim of this resource is to provide teachers strategies to reduce sedentary time and increase PA levels during their academic lessons. A total of 60 short videos recorded in the real context of primary/secondary educational centers were generated, and that represent the examples of activities to implement PAL. The activities were specifically chosen to be relevant for all schools from the participant’s countries, being an easy resource to implement and adaptable to any context. All activities were classified into function of the subject (Math’s, Natural Sciences, Language, and Social Sciences) and the educational stage (primary/secondary) for facilitate their use. Overall, the videos show the initial disposition and the dynamics for the implementation of the activities. Additionally, by opening the videos in *YouTube* platform, teachers can find the necessary materials to put the activity into practice.

***Real time AB platform.*** The goal of this resource was to provide teachers with an easy-to-use digital tool to implement AB during academic lessons. The *Real Time AB Platform* was implemented by involving a virtual avatar that guided all AB and it also allowed customizing the proposals to adapt them to the interests/objectives of the teachers. The avatar allowed communication with the platform users using C1-level translation applications such as *deepl.com* and voice synthesis (*text2speech*). This platform allowed the deployment of:

- *AB without learning/reinforcement academic contents* (the goal is to exercise). The avatar leads AB and students must imitate the movements made by the avatar. The teacher can choose from an available list of the movements/exercises, as well as methods (workout/game models) and difficulty, according to their preferences and objectives of the AB.
- *AB while learning/reinforcing academic contents of different subjects*. Teachers can customize the activities by choosing the content of the question and the result of the action. The content of the question will be voiced by the avatar using the voice synthesis module in the language chosen by the teacher. The teacher will have a list of possible actions to be taken by the students in response to the activities.

Additionally, the platform allows you to make/edit AB designed by the consortium (including AB without learning and AB while learning contents of dietary patterns, SH, and PA) or designed by other users who wish to make their AB public. This characteristic will generate a set of AB in continuous growth with new proposals that will be hosted on the platform, guaranteeing their autonomy and sustainability. The user will be able to access the app online multi-device using either a mobile device or a computer.

***ACS toolkit.*** It is a document with the aims to change the perception of the primary/secondary school community in relation to ACS. This document provides information about strategies to promote ACS for school leaders, teachers, and parents for schoolchildren to increase PA. Moreover, the steps follow to carry out the strategies correctly are explained, and some practical examples are provided in the form of LU. As different perspectives and geographical, cultural, and organizational backgrounds were involved, the tools are relevant to a broad group of stakeholders.

***LU about HL promotion (PA, healthy nutrition, and SH).*** This resource is made up of a set of LU for primary/secondary students that can be put into practice from different subjects. The LU aims to introduce a new approach to learning at school through appropriate strategies resulting in a change in everyday lifestyle and improvement in the life-skills of students, parents, and teachers. The LU was responded to these issues: increasing of PA and reducing sedentary behavior through extra-curriculum and school-based PA; and promoting healthy nutrition, active lifestyle, and healthy SH. This resource included 54LU: 24 units related to healthy nutrition, 10 for SH, and 20 for PA.

***Parents toolkit about promoting HL (PA, healthy nutrition, and SH).*** It is a manual/guide that provides parents with strategies to promote HL concerning the PA, sedentary behaviors, sleep, nutrition, and help them to prepare and implement physical activities suitable to their child-life circumstances.

***School leader’s toolkit.*** This resource was aimed to provide school leaders with a toolkit of strategies and interventions to CAS environment thorough increase PA and reduce sedentary time during the school time (multi-component interventions, environmental modifications in the recess, classroom, etc.).

#### Dissemination plan

The goal of this phase was to carry out the implementation/dissemination of the resources. There were two ways of promotion:

***Online dissemination thought the EUMOVE platform.*** Both the non-governmental organizations and local stakeholders of each country were used their webpages, social networks, and email contact lists to share the EUMOVE platform with schools/teachers. Furthermore, EUROPEACTIVE played an important role in the international dissemination, given the collaboration with national associations from more than 30 European countries. Similarly, HEPA Europe network disseminated the outcomes of the project across European organizations, other projects, and networks around HL promotion. Additionally, we also created an Instagram and X account (@EumoveProject) to promote the project. Specifically, during the three years of the project, we managed to achieve more than 23 500 impressions, more than 1000 profile visits, and more than 80 interactions/day with content.

***Face-to-face dissemination.*** Each public university, with the collaboration of the national non-governmental organizations, hosted a scientific seminar in their country targeted at researchers, teachers, and public administrations. These scientific seminars had the aim of sharing experiences for the promotion of HL from educational centers and promoting the resources. Also, the national non-governmental organizations, in conjunction with the public university’s participants of each country and several schools of primary/secondary, organized different teacher and parent workshops. On the one hand, the objective of the teacher workshops was to share in a practical way the resources created, so that they could implement in the real context. On the other hand, the goal of the parent workshops was to offer families strategies to increase PA, reduce sedentary lifestyle, optimize SH and nutritional patterns of their children, as well as promote the resources of the EUMOVE project. A total of 362 researchers/stakeholders attended the scientific seminars, 400 teachers attended the workshops for teachers, and 130 parents/legal guardians attended the workshops for parents.

## Discussion

This article describes the methods of EUMOVE project aimed at developing and disseminating a set of resources that allow the entire educational community to promote HL in European students.

Given that sedentary time and rates of overweight/obesity have been increasing among European students [1, 5, 6, 30], it is necessary to develop school programs to promote HL [10, 11, 31]. In this line, previous research [16, 17, 32, 33] has suggested that the whole-school approaches with multicomponent strategies are the most effective programs for promoting HL. Echoing these calls, we have developed the first Erasmus+ program to provide a set of resources based on scientific evidence to help schools and school-wide interventions implement a paradigm shift based on the CAS [16] for adequate promotion of HL. Furthermore, following current recommendations [16] to facilitate this change, the tools generated was hosted on a web portal.

The particularities of EUMOVE could lead to establish HL changes (increase PA, reduce sedentary time, and adopt good diet and SH) and, consequently, improve health and prevent the obesity among schoolchildren. This will be possible through the dissemination and implementation in a whole approach of the resources generated to help teachers, parents, school leaders, and stakeholders to improve HL promotion in students from a multi-activity perspective. This strategy allows a diversified and complete approach toward any need present in the different actors involved. Therefore, everyone can find the best ways to apply the project by adapting it to their own characteristics. Indeed, previous studies have shown that it is essential to involve the entire educational community to maximize the effects of school interventions [16, 18]. In this sense, the project also developed workshops in each participating region that provided an opportunity for organizations and individuals attending to reflect on their practices in promoting HL and to become familiar with the EUMOVE teaching resources.

This complete experience will possibly result in actions related with educational, sport, and public health interventions, concerning the promotion of HL in schools using the seven opportunities generated, both in partners’ countries but also in Europe in general. We also expect a direct impact on teaching practices and levels of knowledge about HL promotion. Specifically, the expected impact on school leaders, teachers, and stakeholders is: adopt EUMOVE’s guides and tools; increase teachers’ capability, opportunity, and motivation to adopt/implement PAL, AB, ACS, and LU about HL in school setting; create school environments that facilitate the adoption of HL; and enhance the quality of teacher training courses about HL promotion. Similarly, the expected result on parents adopts EUMOVE’s toolkit for parents and implement strategies that facilitate the adoption of HL among their children. In the case of public health and project partners, we expect: their increased insight and the fostering of relationships toward the professional field and stakeholders in terms of providing a closer relation between society and academia on HL promotion in school setting; increase the number of teachers training organizations across Europe who incorporate PAL, AB, ACS, and LU about HL promotion within teacher training; increase in the size and depth of HL promotion research networks; and promote others international projects.

Consequently, we expect a longer-term impact on behavioral and health outcomes of many students who will be indirect beneficiaries of EUMOVE. Future studies should continue the project through a complex/multidimensional evaluation to test whether these interventions are effective/sustainable over time when developed in real-life conditions.

Some strengths of this study include: EUMOVE is an ecological proposal that engages partners of different countries with expertise in developing innovative resources to promote HL in collaboration with the educational community; educational, political, and social entities have participated in the project, which have allowed a holistic approach to the problem of sedentary lifestyle and obesity; this project offers a wide range of opportunities for the educational community with the aim of addressing this goal from a multi-activity perspective; all resources generated are Open Access and multilanguage, adapted to the study plans of each country, and easy to implement; and the project allows schools/stakeholders to increase teachers’ capability, opportunity and motivation to implement PAL, AB, ACS, and LU about HL through the adoption of the resources generated.

We should recognize some limitations. It is necessary to point out that the main result of EUMOVE was the development/dissemination of different resources based on the CAS framework. Given the nature of the study, no data were obtained in terms of effectiveness with variables such PA, fitness, nutrition, sleep, or body composition. Similarly, a qualitative approach was not considered to understand the perception/attitudes of the different target groups about the experience, functionality, barriers, and facilitators of the tools designed and that, consequently, could improve the project design. Thus, our resources must be used with caution.

In conclusion, this study describes the methods of a pioneering European project for promote HL in the school from a whole and multicomponent approach (the CAS framework) through the design of innovate resources for teachers, parents, school leaders, and stakeholders. The implementation of this toolkit could help the entire educational community to establish HL changes in schoolchildren and improve their health. Future studies should evaluate the acceptability, sustainability, and effectiveness of this proposal.

## Supplementary Material

ckae113_Supplementary_Data

## Data Availability

There are no new data associated with this article. Key points The EUMOVE project follow CAS framework to develop a compressive set of educational resources based of scientific evidence: physically active lessons, active breaks, recess, active commuting to/from school, promoting extracurricular healthy lifestyles, parents, and school environment. All educational resources generated are Open Access and multilanguage, are adapted to the particular study plans of each country and are easy to implement for the users. We used three sources of behaviors (capability, motivation, and opportunity) for implementation across the five target groups (school leaders, teachers, children/adolescents, parents/guardians, and wider stakeholders/policy) through different intervention functions (education, persuasion, enablement, training, modeling, restrictions, and environmental restructuring), by using seven opportunities (the resources designed by the consortium) with the specific goal of increase PA levels, reduce sedentary time, and promote good diet and sleep habits. Educational, political, and social entities have participated in the EUMOVE project, which has allowed a holistic and complementary approach to the problem of sedentary lifestyle and obesity.
