# Supplementary material for: The Erasmus+ EUMOVE project—a school-based promotion of healthy lifestyles to prevent obesity in European children and adolescents
Source: Eur J Public Health. 2024 Jul 29;34(5):955–61. doi: 10.1093/eurpub/ckae113 (PMC11430908; doi:10.1093/eurpub/ckae113)
Supplement: ckae113_Supplementary_Data [file ckae113_supplementary_data.zip › ckae113_Supplementary_Data/ejph-2024-03-om-0172-File006.pdf]

Supplementary Material 1. Main tasks developed in the EUMOVE transnational meetings

**Transnacional Meeting (TM)**	**Tasks**
**TM1: Cáceres, Spain**	Planification about:<br>- Coordination and monitoring of project activities.<br>- Organization of financial processes.<br>- EUMOVE Learning platform.<br>- Planification of the first part of the educational resources.
**TM2: Bologna, Italy**	- Presentation of a preliminary version of all educational resources.<br>- Brainstorming for improving educational resources.
**TM3: Lisbon, Portugal**	- Presentation of the final version of the educational resources.<br>- Definition of dissemination plan.
**TM4: Nice, France**	- Planification of dissemination part.<br>- Analysis of the current status of the project.<br>- Analysis of the impact of the first part of dissemination.<br>- Analysis of events already developed, and planification of those to implement.
**TM5: Cádiz, Spain**	- Final Event.<br>- Presentation of EUMOVE findings and implications and future plans to: HEPA Europe, sport scientists, teachers, parents, educational and local authorities, sport Federations, sport associations...<br>- Planification of the final report for the European Commission.

Abbreviations: HEPA Europe, European network for the promotion of health-enhancing physical activity.
