# Supplementary material for: The Erasmus+ EUMOVE project—a school-based promotion of healthy lifestyles to prevent obesity in European children and adolescents
Source: Eur J Public Health. 2024 Jul 29;34(5):955–61. doi: 10.1093/eurpub/ckae113 (PMC11430908; doi:10.1093/eurpub/ckae113)
Supplement: ckae113_Supplementary_Data [file ckae113_supplementary_data.zip › ckae113_Supplementary_Data/ejph-2024-03-om-0172-File007.pdf]

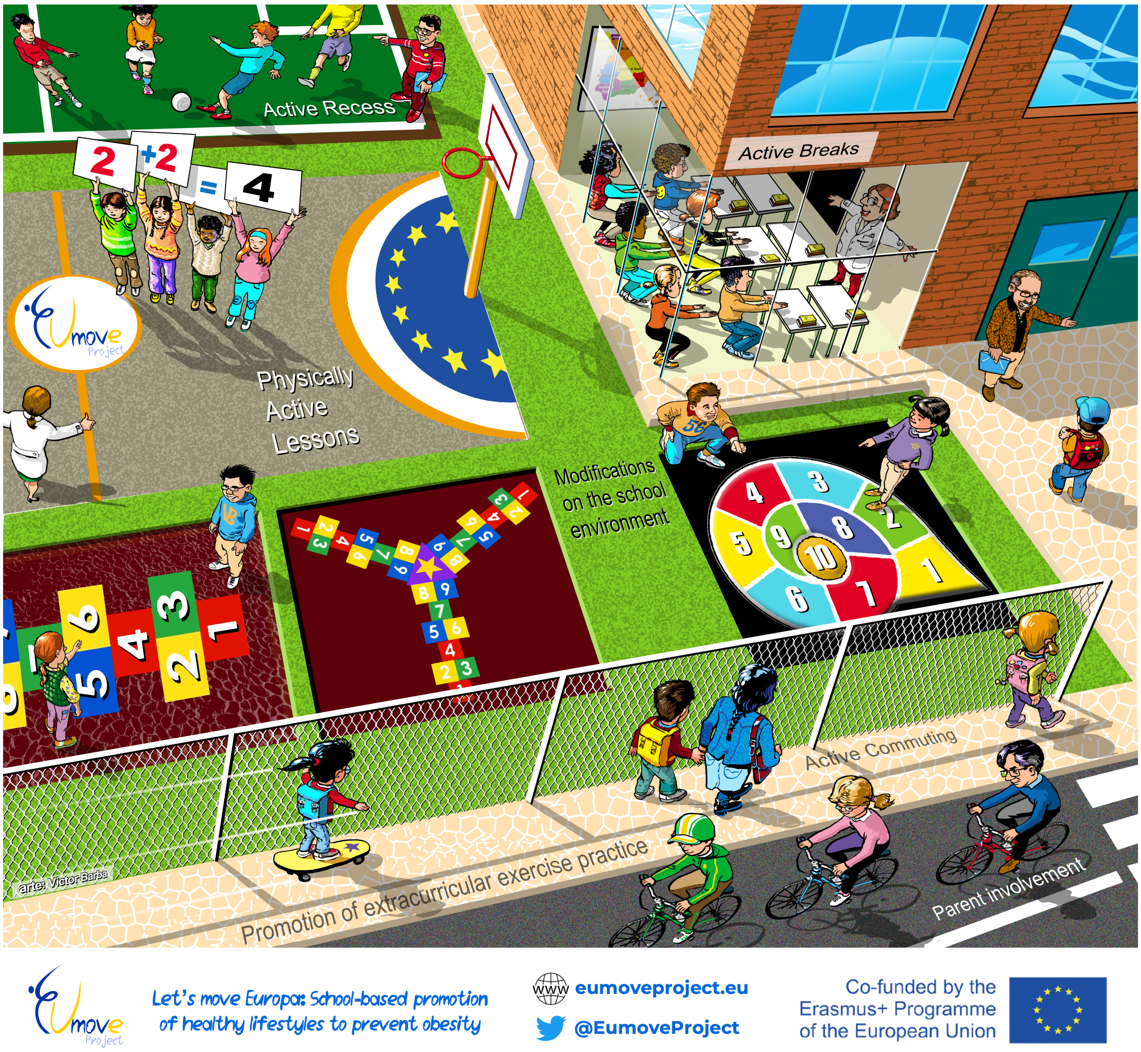

Supplementary Material 2. Infographic of the educational resources generated by the EUMOVE project.
