# Supplementary material for: The Erasmus+ EUMOVE project—a school-based promotion of healthy lifestyles to prevent obesity in European children and adolescents
Source: Eur J Public Health. 2024 Jul 29;34(5):955–61. doi: 10.1093/eurpub/ckae113 (PMC11430908; doi:10.1093/eurpub/ckae113)
Supplement: ckae113_Supplementary_Data [file ckae113_supplementary_data.zip › ckae113_Supplementary_Data/Table S1.docx]

| **Supplementary Material 1.** Main tasks developed in the EUMOVE transnational meetings | |
| --- | --- |
| **Transnacional Meeting (TM)** | **Tasks** |
| **TM1: Cáceres, Spain** | Planification about:  - Coordination and monitoring of project activities.  - Organization of financial processes.  - EUMOVE Learning platform.  - Planification of the first part of the educational resources. |
| **TM2: Bologna, Italy** | - Presentation of a preliminary version of all educational resources.  - Brainstorming for improving educational resources. |
| **TM3: Lisbon, Portugal** | - Presentation of the final version of the educational resources.  - Definition of dissemination plan. |
| **TM4: Nice, France** | - Planification of dissemination part.  - Analysis of the current status of the project.  - Analysis of the impact of the first part of dissemination.  - Analysis of events already developed, and planification of those to implement. |
| **TM5: Cádiz, Spain** | - Final Event.  - Presentation of EUMOVE findings and implications and future plans to: HEPA Europe, sport scientists, teachers, parents, educational and local authorities, sport Federations, sport associations...  - Planification of the final report for the European Commission. |
| Abbreviations: HEPA Europe, European network for the promotion of health-enhancing physical activity. | |
